# Incidence and risk of hypertension in patients newly treated for multiple myeloma: a retrospective cohort study

**DOI:** 10.1186/s12885-016-2955-0

**Published:** 2016-11-22

**Authors:** Ajai Chari, Khalid Mezzi, Shao Zhu, Winifred Werther, Diana Felici, Alexander R. Lyon

**Affiliations:** 1 Icahn School of Medicine at Mount Sinai, 1 Gustave L. Levy Place, New York, NY USA; 2 Amgen Inc., One Amgen Center Dr, Thousand Oaks, CA USA; 3 Simulstat, Inc., 4370 La Jolla Village Dr, San Diego, CA USA; 4 Amgen Inc., 1120 Veterans Blvd, South San Francisco, CA USA; 5 Onyx Pharmaceuticals, Inc., an Amgen subsidiary, 1641 Kansas St, Redwood City, CA USA; 6 NIHR Cardiovascular Biomedical Research Unit, Royal Brompton Hospital and Imperial College London, SW3 6NP, London, UK

**Keywords:** Cardiovascular comorbidity, Hypertension, Incidence, Multiple myeloma, Newly-treated, Risk factor

## Abstract

**Background:**

Hypertension is commonly reported in multiple myeloma (MM) patients and may be associated with older age, disease-related complications and consequences of MM treatments. This study evaluated the incidence rates of and risk factors for hypertension and malignant hypertension in newly-treated MM patients in the United States.

**Methods:**

Newly-treated adult MM patients were identified from Truven MarketScan claims database from 1/1/05 to 3/31/14. Inclusion criteria were new diagnosis of MM with start of MM treatment, ≥12 months continuous enrollment prior to diagnosis, ≥30 days of continuous enrollment following initial diagnosis, and prescription drug coverage. Non-MM patients were matched for age (within +/− 5 years), sex and distribution of index dates to MM patients. Baseline cardiovascular (CV) comorbidities, incidence rate of hypertension and malignant hypertension in the follow-up period, and risk of hypertension and malignant hypertension based on existing baseline CV comorbidities were evaluated.

**Results:**

A total of 7895 MM patients (38% with hypertension history) and 23,685 non-MM patients (24% with hypertension history) were included in the study. Twenty-two percent of MM patients versus 3% of non-MM patients had baseline renal failure. A higher percentage of MM versus non-MM patients had baseline hypertension in combination with renal failure, congestive heart failure or both. The incidence rate of hypertension in MM and non-MM patients was 260 and 178 per 1000 person-years, respectively. There was a 30% increase in the risk of hypertension for MM versus non-MM patients: hazard ratio (HR) 1.30 (95% confidence interval [CI] 1.22, 1.37). In MM patients with a history of hypertension, the risk of malignant hypertension was significantly increased with the following comorbid conditions: cardiomyopathy, HR 2.79 (95% CI 1.20, 6.48); renal failure, HR 2.13 (95% CI 1.36, 3.34); and diabetes mellitus, HR 1.59 (95% CI 1.05, 2.39).

**Conclusions:**

This study confirms that the incidence of hypertension and malignant hypertension is significantly higher in newly-treated MM versus non-MM patients. Hypertension is a risk factor for MM patients developing malignant hypertension. Management of CV comorbidities in MM patients is important based on the increased risk of hypertension and malignant hypertension among patients with these comorbidities.

**Electronic supplementary material:**

The online version of this article (doi:10.1186/s12885-016-2955-0) contains supplementary material, which is available to authorized users.

## Background

Multiple myeloma (MM) is a bone marrow cancer characterized by clonal plasma cells that may lead to anemia, hypercalcemia, renal insufficiency and bone destruction [1]. It is estimated that 30,330 individuals in the United States (US) will be newly diagnosed with MM in 2016, the majority of whom are elderly (50% aged ≥69 years of age) [2]. Even without MM, the elderly population are at an increased risk for development of cardiovascular (CV)-related comorbidities, including hypertension [3]. Given the mean age at diagnosis, complications of MM (eg, bone pain, renal impairment) and frequent use of corticosteroids (with associated weight gain and anxiety), hypertension and malignant hypertension events are likely to occur in patients undergoing therapy for MM.

Epidemiological data on incidence of hypertension in the general population are available; however, very little is currently published on the incidence rates of hypertensive crisis and malignant hypertension in oncology populations, including patients with MM. Taking into consideration patients with MM are often elderly and likely to have pre-existing CV comorbidities and polypharmacy, evaluating the risk factors for hypertension would better enable CV risk management in these patients. This study evaluates the incidence rates of and risk factors for hypertension and malignant hypertension in newly-treated MM patients in the US.

## Methods

### Data source

This retrospective cohort study utilized data from the Truven MarketScan claims database. This database is representative of healthcare received, including treatment patterns and costs of treatment in more than 36 million privately insured patients across the US. It is a fully integrated, patient-level database containing inpatient, outpatient, drug, laboratory, health risk assessment and benefit design information from patients with commercial and Medicare supplemental insurance. MarketScan is compliant with the Healthcare Information Portability and Accountability Act (HIPAA).

### Study population

The study population consisted of newly-treated patients with MM identified from the Truven MarketScan database between January 1, 2005 and March 31, 2014 using the International Classification of Diseases, Revision 9 (ICD-9) codes 203.0, 203.00, 203.01 or 203.02. Patients were included if they were at least 18 years of age and had newly diagnosed MM (one inpatient or two outpatient claims required) with start of MM treatment, ≥12 months of continuous enrollment prior to the first date of MM diagnosis, ≥30 days of continuous enrollment following initial diagnosis, and prescription drug coverage. To exclude monoclonal gammopathy of undetermined significance and asymptomatic myeloma classified as MM, all patients had to be receiving at least one MM drug identified on prescription claims. Patients were excluded if they had another cancer diagnosis within 12 months prior to, or 12 months following the initial MM diagnosis, and received prior chemotherapy.

A non-MM comparator cohort was identified from the original database which included all claims for the MM cohort. Three randomly selected comparator patients with no MM diagnoses between January 1, 2005 and March 31, 2014 were identified for each of the MM patients so the distribution of index dates for the comparators would match those of the MM patients. Non-MM patients were also matched to MM patients on age (within ±5 years of the MM patient’s age at index date) and sex. Non-MM patients included in the study were at least 18 years of age, had continuous enrollment during a 12-month baseline plus at least 1-day follow-up time period, and had annual prescription drug coverage during the year (s) included in the 12 months baseline plus at least 1-day follow-up time period. The only exclusion criteria for non-MM patients was having MM; other non-MM cancers were allowed.

### Study definitions

The index date for MM patients was the date of first treatment claim for MM treatment. The index date for non-MM comparator patients was matched to individuals in the MM group with 365 days of continuous enrollment prior to that date. Baseline was defined as the 365 days of continuous enrollment preceding the index date. Follow-up was defined as the period from index date to first occurrence of an event (first diagnostic code) for those experiencing an event, and was defined as the period from index date to end of enrollment or end of study time period (March 2014) for event-free patients.

### Objectives and study measures

The main objective of this study was to estimate incidence rates of hypertension and malignant hypertension in a representative sample of treated MM patients and non-MM comparator patients in the US. Comparison of hypertensive and malignant hypertensive incidence rates between treated MM patients and non-MM patients, as well as to evaluate the risk of hypertension or malignant hypertension over the follow-up period based on existing hypertension and other baseline CV comorbidities using Cox proportional hazards methods. In addition, the total number of classes of anti-hypertensive medications prescribed at baseline were compared between MM patients and non-MM patients.

### Patient baseline demographics and characteristics

Patient demographics included age, sex, geographic region and calendar year of index date. Hypertensive events were identified from the database using one inpatient or outpatient claim with an ICD-9 code of 401.××, 402.××, 403.××, 404.××, 405.×× or 437.2×. Patients with prior history of hypertension were defined as having a hypertensive event in the baseline period. Other comorbidities included cardiac dysrhythmias, cardiomyopathy, congestive heart failure, ischemic heart disease (acute myocardial infarction and angina), acute myocardial infarction, cerebrovascular disease (hospitalized stroke and transient cerebral ischemia), renal failure, diabetes mellitus, amyloidosis and hyperlipidemia. All comorbidities were identified using one inpatient or outpatient claim (ICD-9 codes; see Additional file 1 Table S1), with the exception of cerebrovascular disease, which was identified using inpatient claims only. The Charlson comorbidity index (CCI) was calculated according to the Quan adaptation [4]. Baseline anti-hypertensive medications by drug class for treatment of hypertension were identified and included diuretics, angiotensin-converting enzyme inhibitors (ACE-I), angiotensin II blockers, calcium channel blockers and other (alpha blockers, alpha-2 receptor agonists, beta blockers, central agonists, combined alpha and beta blockers, peripheral adrenergic inhibitors, renin inhibitors and vasodilators). Baseline anti-hypertensive medications were defined as treatments prescribed in the 3 months before the index date.

### Follow-up period measures

The hypertensive events were identified as described for the baseline period. Malignant hypertensive events were identified using one inpatient claim with an ICD-9 code of 437.2×, 401.0×, 402.0×, 403.0×, 404.0× or 405.0×. The addition of anti-hypertensive medications in the follow-up period was compared between MM patient and non-MM patients. For patients with incident hypertension, anti-hypertensive medications were defined as drugs prescribed after hypertension diagnosis.

### Statistical analyses

Incidence rates were estimated using traditional methods and presented per 1000 person-years (PYRs) with a 95% confidence interval (CI) of any event. A patient was counted in the numerator of the incidence rate at the time of the first diagnostic code for the event in the follow-up period. The risk of hypertension and malignant hypertension (overall and in patients with and without a prior hypertensive event) in the MM and non-MM patients was compared using the Cox proportional hazards regression model. Univariate Cox models were first conducted to assess whether individual baseline variables predicted hypertension or malignant hypertension. Multivariate Cox models were then applied. Age, sex and geographic region were locked into the model and stepwise methods were used to determine which baseline comorbidities to include in the model. Analyses were conducted using SAS® 9.3 (SAS Institute Inc., Cary, NC, USA). Where appropriate, significance was assessed at the *p* < 0.05 level.

## Results

**Fig. 1:**
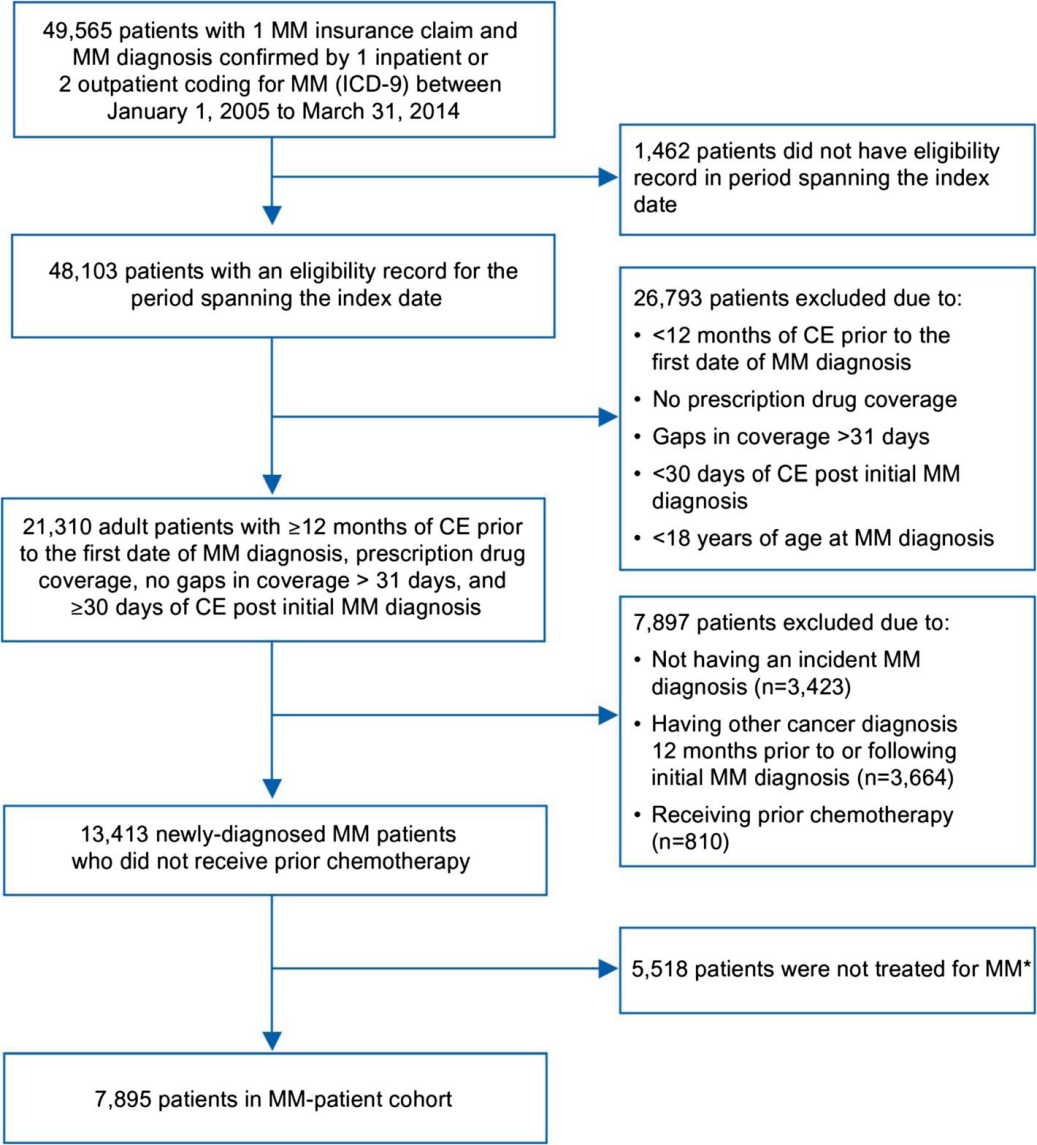
Selection of MM patient cohort. MM drugs identified on prescription claims. CE, continuous enrollment; ICD-9, international classification of diseases, ninth revision; MM, multiple myeloma

A total of 49,565 patients with a MM diagnosis code claim were identified between January 1, 2005 and March 31, 2014 (Fig. 1). Based on inclusion and exclusion criteria, 7895 patients were included in the MM patient cohort for study analysis. A total of 23,685 patients were identified and matched to the MM patients and comprised the non-MM patient cohort for study analysis. The MM and non-MM patients were generally well-matched on distribution of index dates (Table 1).

**Table 1:** Baseline demographics and characteristics

Patient characteristics	MM patients (*n* = 7895)	Non-MM patients (*n* = 23,685)
Duration of follow-up, years		
Median (range)	2 (0–9)	2 (0–10)
Age at index date, years		
Mean ± SD	65.3 ± 11.8	65.3 ± 11.8
Median (range)	64 (18–97)	64 (18–97)
Age, years, *n* (%)		
18–34	26 (0.3)	78 (0.3)
35–44	239 (3.0)	717 (3.0)
45–54	1130 (14.3)	3390 (14.3)
55–64	2747 (34.8)	8241 (34.8)
65–74	1812 (23.0)	5436 (23.0)
75+	1941 (24.6)	5823 (24.6)
Sex, n (%)		
Male	4400 (55.7)	13,200 (55.7)
Female	3495 (44.3)	10,485 (44.3)
Year of index date, *n* (%)		
2005	634 (8.0)	1591 (6.7)
2006	639 (8.1)	1464 (6.2)
2007	607 (7.7)	1492 (6.3)
2008	892 (11.3)	2154 (9.1)
2009	1136 (14.4)	3193 (13.5)
2010	832 (10.5)	2445 (10.3)
2011	1007 (12.8)	3644 (15.4)
2012	1102 (14.0)	4177 (17.6)
2013	908 (11.5)	3039 (12.8)
2014	138 (1.7)	486 (2.1)
Comorbidities at baseline, *n* (%)		
Hypertension	3002 (38.0)	5750 (24.3)
Renal failure	1698 (21.5)	696 (2.9)
Hyperlipidemia	1399 (17.7)	3712 (15.7)
Diabetes mellitus	1242 (15.7)	3007 (12.7)
Ischemic heart disease	841 (10.7)	1777 (7.5)
Cardiac dysrhythmias	563 (7.1)	1019 (4.3)
Congestive heart failure	526 (6.7)	549 (2.3)
Cardiomyopathy	168 (2.1)	176 (0.7)
Amyloidosis	110 (1.4)	3 (0.01)
Acute myocardial infarction	106 (1.3)	133 (0.6)
Cerebrovascular disease^a^	100 (1.3)	159 (0.7)
Hypertension + renal failure	1034 (13.1)	494 (2.1)
Hypertension + congestive heart failure	322 (4.1)	320 (1.4)
Hypertension + renal failure + congestive heart failure	179 (2.3)	116 (0.5)
CCI		
Mean ± SD	1.44 ± 1.93	0.41 ± 1.01
Median (range)	1 (0–12)	0 (0–15)

*CCI*: Charlson comorbidity index, *MM*: multiple myeloma, *SD*: standard deviation

^a^Based on inpatient claim only

The baseline demographics and characteristics for MM patients and non-MM patients are shown in Table 1. Both cohorts were equally matched for sex (55.7% males) and age, with median age (range) at index date of 64 (18–97) years. A total of 49% of patients were 45–64 years of age and 47% were ≥65 years of age (47%); less than 4% were <45 years of age. The median duration of follow-up for both the MM and non-MM patients was 2 years. The percentage of MM patients with baseline CV comorbidities was higher than that among non-MM patients for each of the comorbidities evaluated (Table 1). Hypertension was the most common comorbidity in both groups, with 38% of the MM patients (3002/7895) having hypertension at baseline compared with 24% of the non-MM patients (5750/23,685). Heart failure at baseline was observed in 6.7% (526/7895) and 2.3% (549/23,685) of patients in the MM and non-MM cohorts, respectively. The largest numeric difference between MM and non-MM patients was for presence of baseline renal failure: 22% of MM patients compared with 3% of non-MM patients. A total of 13.1% (1034/7895) of MM patients had both hypertension and acute renal failure at baseline compared with 2.1% (494/23,685) of non-MM patients. A total of 4.1% of MM patients had both hypertension and congestive heart failure at baseline versus 1.4% of non-MM patients. The percentage of patients having hypertension, renal failure and congestive heart failure at baseline was 2.3% for MM patients and 0.5% for non-MM patients. Ischemic heart disease, diabetes mellitus and hyperlipidemia were present at baseline in 11%–18% of MM patients, with corresponding rates for non-MM patients ranging from 8%–16%. Median CCI was 1 (range, 0–12) for the MM patients and 0 (range, 0–15) for the non-MM patients.

### Hypertension in follow-up period

**Fig. 2:**
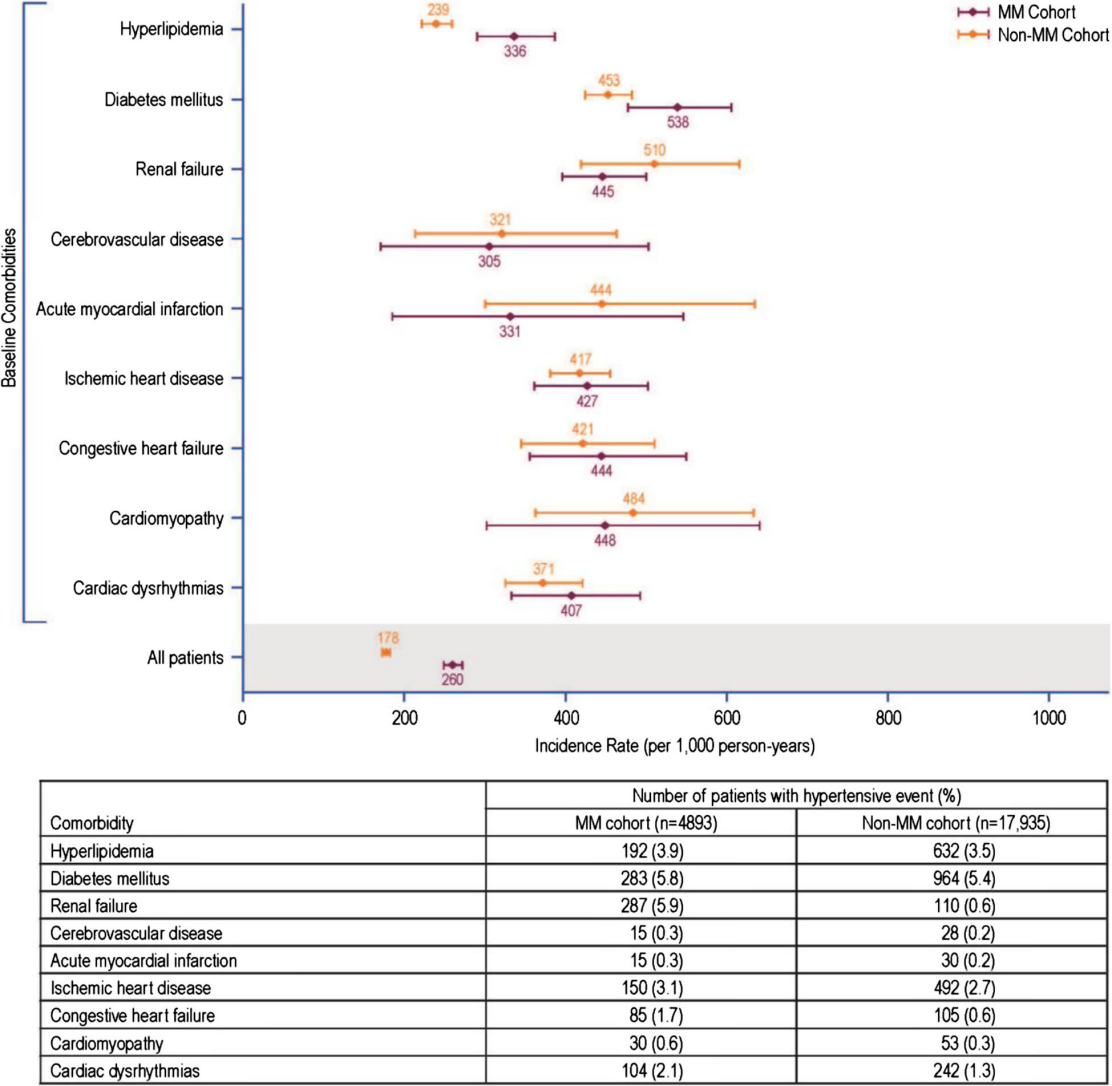
Incidence rate of hypertension (per 1000 PYRs) and 95% confidence intervals. MM, multiple myeloma; PYRs, person-years

The incidence rate of hypertension per 1000 PYRs in patients with MM was 260 (95% CI 248, 272) and in non-MM patients was 178 (95% CI 173, 182) (Fig. 2). Hypertension incidence rates per 1000 PYRs (95% CI) in MM and non-MM patients by baseline comorbidities are shown in (Fig. 2).

### Malignant hypertension in follow-up period

**Fig. 3:**
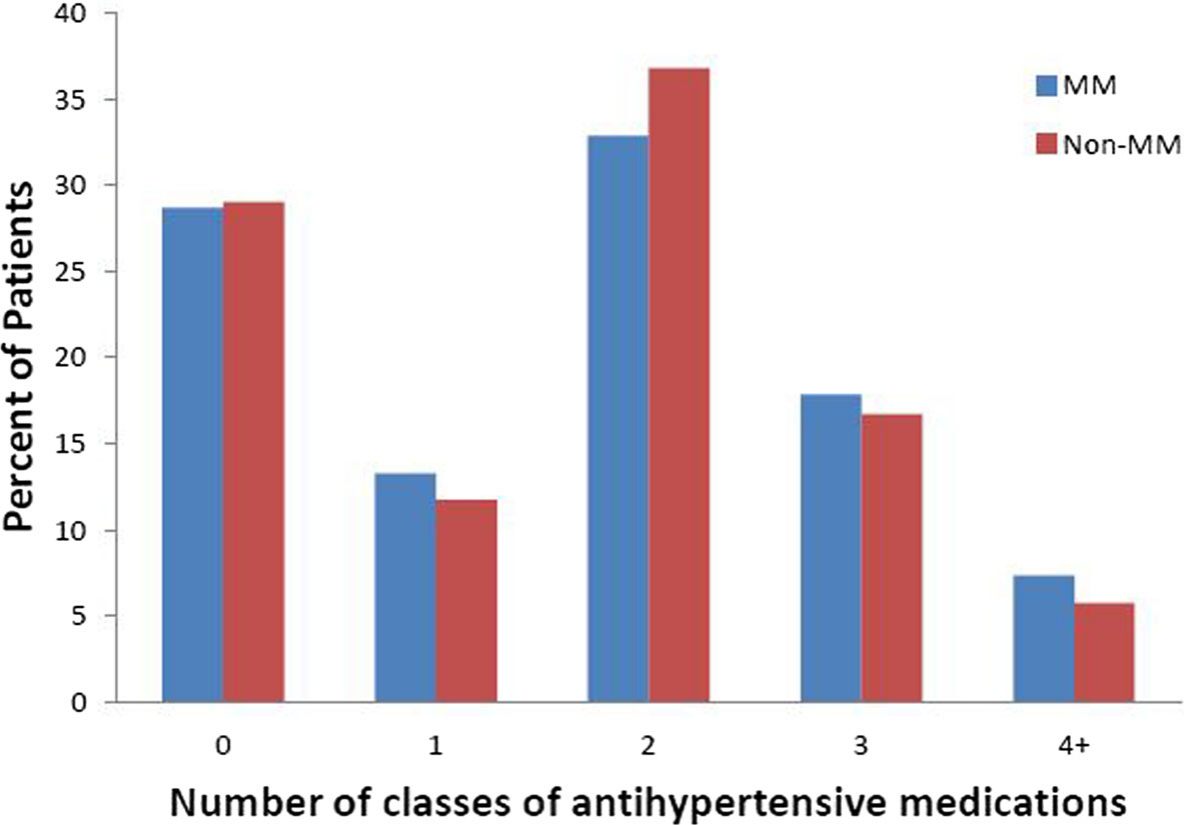
Number of classes of anti-hypertensive medications at baseline for MM and non-MM patients. Anti-hypertensive medications by class included diuretics, ACE-I, angiotensin II blockers, calcium channel blockers and others (alpha blockers, alpha-2 receptor agonists, beta-blockers, central agonists, combined alpha and beta blockers, peripheral adrenergic inhibitors, renin inhibitors and vasodilators). ACE-I, angiotension-converting enzyme inhibitor; MM, multiple myeloma

The incidence rate per 1000 PYRs (95% CI) of malignant hypertension in MM-treated patients without a history of hypertension was 3.3 (95% CI 2.3, 4.5) and in non-MM patients without a history of hypertension was 1.9 (95% CI 1.5, 2.3). In patients with a prior history of hypertension, the incidence rate per 1000 PYRs (95% CI) for malignant hypertension in MM-treated patients was 10.3 (95% CI 7.8, 13.2) and in non-MM patients was 4.3 (95% CI 3.2, 5.5). Rates by baseline comorbidities are shown in (Fig. 3).

### Risk of hypertension or malignant hypertension: Cox proportional hazards modeling

Adjusted hazard ratios (HRs) for hypertension events from the multivariate Cox proportional hazards modeling are presented in Table 2. There was a 30% increase in the risk of hypertension in MM versus non-MM patients. In patients with baseline ischemic heart disease, renal failure, diabetes or hyperlipidemia, the risk of incident hypertension was significantly increased compared with patients who did not have these comorbidities at baseline (*p* < 0.001 for all comparisons; Table 2). Older age (≥55 years) also increased the risk of incident hypertension. MM-treated patients with or without a history of hypertension had a significantly higher risk of malignant hypertension during the follow-up period compared with non-MM patients (previous history of hypertension: HR 1.90, 95% CI 1.26, 2.87, *p* < 0.01; no prior history of hypertension: HR 1.54, 95% CI 1.04, 2.28, *p* < 0.05). In patients with a prior history of hypertension, presence of cardiomyopathy, renal failure or diabetes at baseline significantly increased the risk of malignant hypertension compared with absence of these comorbidities at baseline (*p* < 0.05 all comparisons). In patients without a prior history of hypertension, only age ≥65 years versus 18–54 years and mild or high CCI versus low CCI at baseline was associated with an increased risk of malignant hypertension in the follow-up period (*p* < 0.05 all comparisons).

**Table 2:** Multivariable Cox proportional hazards model: Predictors of hypertension

Predictor	Level	HR (95% CI)	*p*-value
Patient cohort (reference: non-MM patients)	MM patients	1.30 (1.22, 1.37)	<0.0001
Age (reference: 18–54 years)	55–64 years	1.82 (1.69, 1.97)	<0.0001
	65–74 years	2.55 (2.36, 2.76)	<0.0001
	75+ years	2.88 (2.66, 3.11)	<0.0001
Comorbidities at baseline (yes vs. no)	Ischemic heart disease	1.29 (1.20, 1.40)	<0.0001
	Renal failure	1.43 (1.27, 1.61)	<0.0001
	Diabetes mellitus	1.72 (1.59, 1.86)	<0.0001
	Hyperlipidemia	1.16 (1.07,1.26)	<0.0001
CCI (reference: low [0])	Mild (1,2)	1.11 (1.03, 1.20)	<0.01

*CCI* Charlson comorbidity index, *HR* hazard ratio, *MM* multiple myeloma

### Anti-hypertensive medications in MM and non-MM patients

**Fig. 4:**
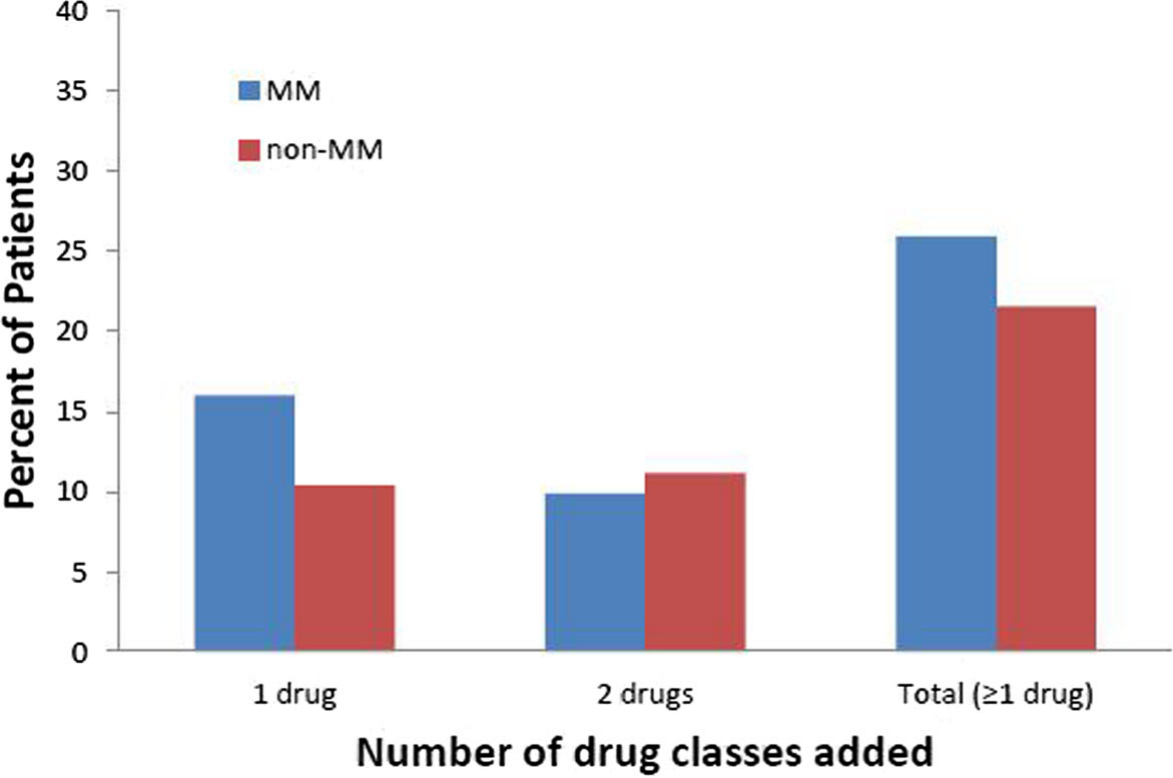
Addition of anti-hypertensive medications during the follow-up period for MM and non-MM patients. Classes of anti-hypertensive medications added included diuretics, ACE-I, angiotensin II blockers, calcium channel blockers and other (alpha blockers, alpha-2 receptor agonists, beta-blockers, central agonists, combined alpha and beta blockers, peripheral adrenergic inhibitors, renin inhibitors and vasodilators). ACE-I, angiotension-converting enzyme inhibitor; MM, multiple myeloma

The numbers of MM and non-MM patients taking anti-hypertensive medications at baseline are shown in Table 3. The proportion of patients receiving at least one class of anti-hypertensive medication at baseline was the same for MM and non-MM patients (71%). The number of classes of anti-hypertensive medication at baseline between the two groups was similar (Fig. 3). Among patients who were treated for hypertension, the most common medications at baseline for both groups were diuretics, ACE-I, calcium channel blockers and angiotensin II receptor blockers (ARBs) (Table 3). For patients with incident hypertension, 1425 of 1865 (76.4%) MM patients and 4548 of 5861 (77.6%) non-MM patients received at least one class of anti-hypertensive medication during follow-up. A total of 16.0% of MM patients and 10.4% of non-MM patients received one new class of anti-hypertensive medication during the follow-up period; 9.9% of MM patients and 11.2% of non-MM patients received two additional classes of anti-hypertensive medications during the follow-up period (Fig. 4).

**Table 3:** Baseline anti-hypertensive drugs in patients with a history of hypertension

	MM Patients (*n* = 3002)	Non-MM Patients (*n* = 5750)
Anti-hypertensive drug, n (%)		
All drugs^a^	2141 (71%)	4082 (71%)
Diuretic	1704 (80%)^b^	3339 (82%)^b^
ACE-I	1113 (52%)^b^	2180 (53%)^c^
Calcium channel blocker	1081 (50%)^b^	1773 (43%)^c^
ARB	914 (43%)^b^	1723 (42%)^c^
Any other drugs	53 (2%)^b^	102 (2%)^c^

*ACE-I*: angiotensin-converting enzyme inhibitor, *ARB*: angiotensin II receptor blocker, *MM*: multiple myeloma

^a^All anti-hypertensive drugs included diuretics, ACE-I, ARBs, calcium channel blockers and other (alpha blockers, alpha-2 receptor agonists, beta-blockers, central agonists, combined alpha and beta blockers, peripheral adrenergic inhibitors, renin inhibitors and vasodilators)

^b^Percentage derived from *n* = 2141 MM patients treated for hypertension

^c^Percentage derived from *n* = 4082 non-MM patients treated for hypertension

## Discussion

Hypertension is commonly reported in patients with MM in clinical trials and may be associated with older age, disease-related complications or consequence of MM treatments [5–7]. However, little is known about the incidence of hypertension or malignant hypertension in the broader population of patients outside of clinical trials. To our knowledge, this is the first study to estimate the incident rates of hypertension and malignant hypertension in a population of newly-diagnosed, MM-treated patients in the US not participating in clinical trials compared with age- and gender-matched non-MM patients. There is a recent study by Kistler and colleagues that reported incidence rates of hypertension in combination with arterial events as part of their evaluation of cardiac events in MM and non-MM patients based on MarketScan data [8]. They found no significant difference in risk of hypertension/arterial events between MM and non-MM patients. This differs from the results of this current study which found a 30% higher risk of hypertension in MM patients versus non-MM patients. There were a couple of significant differences in study design between the two studies. Kistler et al. did not specifically study hypertension events alone; they evaluated the incidence of hypertensive and arterial events combined. In addition, the MM patients in the Kistler study had a longer duration of MM disease, as the inclusion criteria for the MM cohort required patients to have had at least three anti-myeloma treatments (thereby introducing confounding medical issues such as autonomic or adrenal insufficiency, weight loss, etc.), whereas the current study included newly-diagnosed MM patients with at least one anti-myeloma treatment.

In this study, the prevalence rate of hypertension in non-MM patients (33% of patients) is comparable with published data for the US adult population (1 out of 3 adults) [9, 10]. That said, the incidence of hypertension and malignant hypertension is significantly higher in newly-treated MM patients compared with non-MM patients. Multi-variate analyses showed that patients with MM had a statistically significant increased risk of hypertension compared with non-MM patients and also a significantly increased risk of malignant hypertension in both MM patients with or without a history of hypertension compared with non-MM patients. Whether the increased risks of hypertension and malignant hypertension found for MM patients were due to disease-unrelated factors, disease-related comorbidities or a combination of these factors is difficult to determine. Older age and male gender pre-disposes MM patients to an increased risk of hypertension; however, this study controlled for both these factors by using age- and sex-matched non-MM patients.

Results of multi-variable modeling found that the presence of several CV comorbidities increased the risk of hypertension and malignant hypertension in MM patients. In patients without a prior history of hypertension, co-existing ischemic heart disease, renal failure, diabetes and hyperlipidemia increased the risk of hypertension. In patients with a prior history of hypertension, co-existing cardiomyopathy, renal failure or diabetes greatly increased the risk of malignant hypertension. The presence of all of these co-morbidities was significantly higher in the MM population than in the non-MM population at baseline. High levels of CV comorbidities in MM patients have been noted in another non-clinical study of newly-diagnosed MM patients. Chen et al. reported that close to half (47.9%) of all newly-diagnosed MM patients (*N* = 8239) identified from commercial medical and Medicare claims databases had more than one type of comorbidity at baseline (6 months prior to MM diagnosis), with 43.9% of patients having metabolic comorbidities, 21.4% with CV diseases and 11.5% with renal conditions [11].

Renal dysfunction is very common in MM patients and renal failure is a negative prognostic factor for patient survival [5]. In this study, renal failure at the start of treatment in patients without a history of baseline hypertension did appear to be a risk factor for hypertension during treatment; however, patients were only at risk for malignant hypertension if renal failure was also associated with baseline hypertension.

Hypertension has been reported to be twice as frequent in patients with diabetes than in those without diabetes [12]. The findings from this study also show close to double the rate of hypertension in non-MM patients with diabetes (54%) versus those without diabetes (30%) (data not shown). For MM patients, the rate of hypertension was 54% in those with co-existing diabetes and 36% in patients without diabetes (data not shown). This is of particular concern given that the routine use of corticosteroids in myeloma therapy can lead to new diagnoses of diabetes or worsen glycemic control of those with known diabetes. Taken together, these results show that control and prevention of hypertensive events in MM patients must include management of CV comorbidities.

Per the 8th Joint National Committee of 2014 evidence-based guidelines for management of high blood pressure in adults, “hypertension is one of the most important preventable contributors to disease and death” [13]. The guidelines recommend initiating drug treatment in non-black hypertensive patients with an ACE-I, ARB, calcium channel blocker or thiazide-type diuretic; in black hypertensive patients, initial therapy should include a calcium channel blocker or thiazide-type diuretic. The most common anti-hypertensive medications at baseline for this study were diuretics, ACE-I, calcium channel blockers and ARBs. For patients with a history of hypertension, the same percentage of patients (71%) in the MM and non-MM groups were receiving anti-hypertensive medications, as well as similar numbers of anti-hypertensive medications at baseline. During the follow-up period, similar percentages were seen between the MM patients and non-MM patients with incident hypertension.

The choice of anti-hypertensive therapy in a myeloma patient, however, must take into account myeloma-associated renal failure (hence caution with diuretics and ACE-I/ARB), hypercalcemia or hyperuricemia (which can be exacerbated by thiazides), and steroid-related edema (which can be exacerbated by calcium channel blockers). It should also be noted that grade 3 hypertension in clinical trials does not necessarily equate to markedly elevated blood pressures or malignant hypertension, as the addition of blood pressure medications is considered a grade 3 hypertension adverse event per National Cancer Institute Common Toxicity Criteria definition. Addition of anti-hypertensive medications was not included as a hypertensive event in this study; however, evaluation of anti-hypertensive medications in the follow-up period found approximately 6% more MM patients than non-MM patients had one class of anti-hypertensive medication added.

The results of this study emphasize that CV and hypertensive adverse effects cannot be evaluated in clinical trials without a comparator arm, given the high incidence rates of these complications in MM patients. MM patients entering the trials are already at a high risk for hypertensive events, and existing hypertension is a major risk factor for development of malignant hypertension. Hypertension has been reported as an adverse event in studies of patients undergoing MM treatment [5–7, 14–18].

Although this study evaluated hypertension and malignant hypertension in MM patients undergoing treatment for MM, it did not evaluate results for specific anti-myeloma treatments. There are some other limitations of this study. MarketScan claims database better represents the demographic distribution of employed populations while under-representing the elderly, unemployed and disabled. This may be a reason why the median age of MM diagnosis in this study, 65 years, was close to, but a little younger than that published in the literature for median age of MM incidence (69 years SEER cancer statistics) [2]. The MarketScan database does not include information about race, precluding examining the effect of race on these findings (for example, hypertension rates in black vs. white patients, since MM is two-fold more common than in white patients) [2]. In addition, survival data and hypertension and other CV co-morbidity risk factors such as obesity, diet, physical activity and smoking status are not included in the database. There exists the possibility that malignant hypertension may be underestimated in MM patients for whom hospital admissions were not explicitly coded as such, due to the presence of other acute medical issues such as disease progression. Finally, this study could not control for ascertainment bias; MM patients under treatment would be evaluated more frequently by physicians than non-MM patients and thus have a higher probability of hypertensive events reported.

## Conclusion

The incidence of hypertension and malignant hypertension is significantly higher in newly-treated MM patients compared with non-MM patients. Hypertension is a risk factor for MM patients developing malignant hypertension. The presence of hypertension and co-existing cardiomyopathy, renal failure or diabetes also significantly increase the risk of MM patients developing malignant hypertension. Due to the introduction of novel efficacious agents that will likely improve life expectancy, more MM patients will be living longer and will likely be at a greater risk of developing CV complications. The findings of this study highlight the need for a multi-disciplinary approach in managing MM, especially in elderly patients at a greater risk of CV events. A close collaboration between oncologists, cardiologists, nephrologists and primary care physicians is warranted.

## Additional file

Additional file 1: Table S1. Comorbidity ICD-9 codes. ICD-9, International Classification of Diseases, ninth revision. (DOCX 25 kb)

## Abbreviations

ACE-I: Angiotensin-converting enzyme inhibitor
ARB: Angiotension II receptor blocker
CCI: Charlson comorbidity index
CI: Confidence interval
CV: Cardiovascular
HIPAA: Healthcare Information Portability and Accountability Act
HR: Hazard ratio
ICD-9: International Classification of Diseases, Revision 9
MM: Multiple myeloma
PYR: Person year
SD: Standard deviation
US: United States
