# Supplementary material for: Incidence and risk of hypertension in patients newly treated for multiple myeloma: a retrospective cohort study
Source: BMC Cancer. 2016 Nov 22;16:912. doi: 10.1186/s12885-016-2955-0 (PMC5120425; doi:10.1186/s12885-016-2955-0)
Supplement: Additional file 1: Table S1. — Comorbidity ICD-9 codes. ICD-9, International Classification of Diseases, ninth revision. (DOCX 25 kb) [file 12885_2016_2955_MOESM1_ESM.docx]

**Appendix**

**Supplementary Table 1** Comorbidity ICD-9 codes

| **Comorbidities** | **ICD-9 codes** |
| --- | --- |
| Cardiac dysrhythmias | 427.0, 427.1, 427.2, 427.3x, 427.4x, 427.5, 785.0 |
| Cardiomyopathy | 425.x |
| Congestive heart failure | 398.91, 402.01, 402.11, 402.91, 404.01, 404.11, 404.91, 404.03, 404.13, 404.93, 428.xx |
| Ischemic heart disease | 410.xx, 411.1x, 411.xx, 413.xx, 414.xx |
| Acute myocardial infarction | 410.xx |
| Cerebrovascular disease (stroke) | 430, 431, 432, 433.x1, 434.x1, 435.x |
| Renal failure | 403.xx, 404.xx, 582.xx, 583.xx, 584.x, 585.xx, 586, 588.xx |
| Diabetes mellitus | 250.xx |
| Amyloidosis | 277.3, 277.30, 277.39 |
| Hyperlipidemia | 272.0–272.4 |

ICD-9, International Classification of Diseases, ninth revision
